# Content and system development of a digital patient-provider communication tool to support shared decision making in chronic health care: InvolveMe

**DOI:** 10.1186/s12911-020-1065-8

**Published:** 2020-03-04

**Authors:** Berit Seljelid, Cecilie Varsi, Lise Solberg Nes, Aud-E Stenehjem, Jens Bollerslev, Elin Børøsund

**Affiliations:** 1 Department of Digital Health Research, Division of Medicine, Oslo University Hospital, Oslo, Norway; 2 Institute of Clinical Medicine, Faculty of Medicine, University of Oslo, Oslo, Norway; 3 Department of Psychiatry & Psychology, College of Medicine & Science, Mayo Clinic, Rochester, MN USA; 4 Department of Nephrology, Division of Medicine, Oslo University Hospital, Oslo, Norway; 5 Section of Specialized Endocrinology, Department of Endocrinology, Morbid Obesity and Preventive Medicine, Division of Medicine, Oslo University Hospital, Oslo, Norway

**Keywords:** Shared decision making, eHealth, Digital communication, Patient-provider communication, Digital assessment, Secure messaging, Patient-provider interaction, Patient centered care, Renal transplant recipients, Non-functioning pituitary adenoma

## Abstract

**Background:**

Chronic conditions present major health problems, affecting an increasing number of individuals who experience a variety of symptoms that impact their health related quality of life. Digital tools can be of support in chronic conditions, potentially improving patient-provider communication, promoting shared decision making for treatment and care, and possibly even improving patient outcomes. This study aimed to develop a digital tool for patient-provider communication in chronic health care settings and describes the data collection and subsequent content and software development of the *InvolveMe* tool. *InvolveMe* will provide patients with the opportunity to report symptoms and preferences to their health care providers (HCP), and to use secure messaging to interact with the HCPs.

**Method:**

The study employed a combination of interviews with patients with chronic conditions and focus groups with HCPs, examining experiences with chronic conditions and the potential use of a digital tool for support. Participants were recruited from two outpatient clinics at a university hospital. Data collected from interviews and focus groups were analysed using thematic analysis. Content and software development was informed by the data collection and by tool development workshops.

**Results:**

Analyses from interviews with patients (*n* = 14) and focus groups with HCPs (*n* = 11) generated three main themes: 1) *Making symptoms and challenges visible*, 2) *Mastering a new life,* and 3) *Digital opportunities for follow-up.* Each main theme generated separate subthemes. Theme 1 and 2 gave input for content development of the symptom and needs assessment part of the tool, while theme 3 provided ideas for the software development of the *InvolveMe* tool. Tool development workshops with patients (*n* = 6) and HCPs (*n* = 6) supplemented the development.

**Conclusions:**

A digital tool such as *InvolveMe* has the potential to support shared decision making for patients with chronic health conditions. Through integration with an existing patient portal such a tool can provide opportunities for meaningful interactions and communication between patients and HCP’s, particularly with regards to symptoms, needs and preferences for care.

## Background

A growing number of patients are living with chronic health conditions that affect physical, psychological, social and role functioning, as well as general well-being [1–4]. Patients experience difficulties in recognition of symptoms [5] and that their symptoms often are unknown to health care providers (HCPs) [6, 7]. Comparison between symptoms reported by patients and HCPs has shown that patients detect symptoms sooner [8, 9], and that HCPs often underestimate symptom intensities [8–11]. Experiences of poor communication between patient and HCPs are also common [5, 12]. In addition, patients’ comprehension of health information [12], incorrect beliefs and assumptions, are factors that can interfere with symptom management and seeking help [5]. These issues and challenges call for an improvement of chronic health care.

Routine collection of patient reported outcome measures can enhance the quality of visits and has the potential for improved symptom control and patient satisfaction [13]. A better understanding of health related quality of life (HRQoL) aspects could also enable HCPs to deliver more patient-centered care and thereby have a stronger impact on the overall health and symptom burden experienced by patients [14].

The treatment goal for patients in chronic health care settings is related to adequate symptom control and being able to live with the condition in acceptable ways [15]. Patients have an important role in controlling and self-administering treatment, monitoring symptoms and choosing lifestyle [15], which underlines the importance of patients being involved in their own care. Shared Decision Making (SDM) is considered particularly useful in the management of chronic health conditions, as SDM focuses on engaging patients in their own health care, recognizing that behavioral, psychosocial and lifestyle aspects can affect health beyond biomedical interventions [16]. SDM can be explained as a process where patients and HCPs work together to understand and address the patient’s situation [17]. In a framework for SDM in chronic health care, there is an emphasis on the patient-provider partnership as well as focus on information exchange, choice deliberation and decision making [15].

Establishing trust and mutual respect is crucial for a patient-provider relationship, and a patient-provider relationship includes emotional as well as cognitive care [18], which again may promote problem-solving, communication and support [15]. Patients sharing personal information about symptoms and needs, including values, preferences and expectations, are essential for the HCP to gain an understanding of the patients’ current situation [15].

SDM-tools have been shown to improve patient knowledge, help patients to become more aware of what matters most to them, and help patients to feel more involved in the decision making process [19]. Despite the fact that symptom management is an important and integral part of chronic health care, only few existing SDM-tools include symptom management [19, 20]. Furthermore, a recent systematic review examining SDM tools in chronic health conditions revealed that the included tools were mostly designed to “transfer information” about options and the harms and benefits of these [20]. Suggestions have been made, stating that SDM needs to focus on creating patient-provider interactions promoting communication and care, not just on offering information and choice [17, 21]. This supports the exploration of new ways to promote patient involvement in chronic health care settings, emphasizing patient-provider communication related to symptoms and needs.

Digital systems may carry the potential to aid in SDM in chronic health settings, to address the individuality and variability in symptoms over time among patients, and to improve patient outcomes [14, 22, 23]. Existing research has shown how digital systems can help patients to communicate symptoms [24, 25], to reduce symptom distress and improve overall symptom management [14, 25, 26], as well as to improve HRQoL [14]. Such systems can also help clinicians to provide individually tailored support and increase patient involvement [14, 25–28]. One example of such a digital system is the *Choice*-application, originally developed to support cancer patients with symptom reporting in preparation for consultation [24, 26, 29, 30], subsequently aiding HCPs in individualizing patient care. Other application (app)-based systems designed for self-management in long-term conditions have also been found to improve symptom management, but few existing systems provide features for data sharing ahead of hospital visits [31]. Benefits to patient-provider communication from using secure messaging have also been reported, particularly in terms of assisting patients in self-management of illness and in improving health outcomes [23, 25, 28, 32]. Findings on digital communication systems so far are promising, and such systems can facilitate new ways to promote patient involvement in symptom management of chronic health conditions.

The current study aimed to map patients’ (renal transplant recipients (RTX) or patients with a non-functioning pituitary adenoma (NFPA)) symptoms and needs in preparation for the development of a new digital patient-provider communication tool called *InvolveMe,* and then to design and develop the new tool, tailored to suit each patient’s situation and preferences with regards to symptom management in out-patient chronic health care settings. The goal was to develop and design the *InvolveMe* tool to provide patients with the opportunity to: a) self-report symptoms and preferences for care prior to visits, and b) use secure messaging for patient-provider communication between visits.

## Methods

### Design

**Fig. 1:**
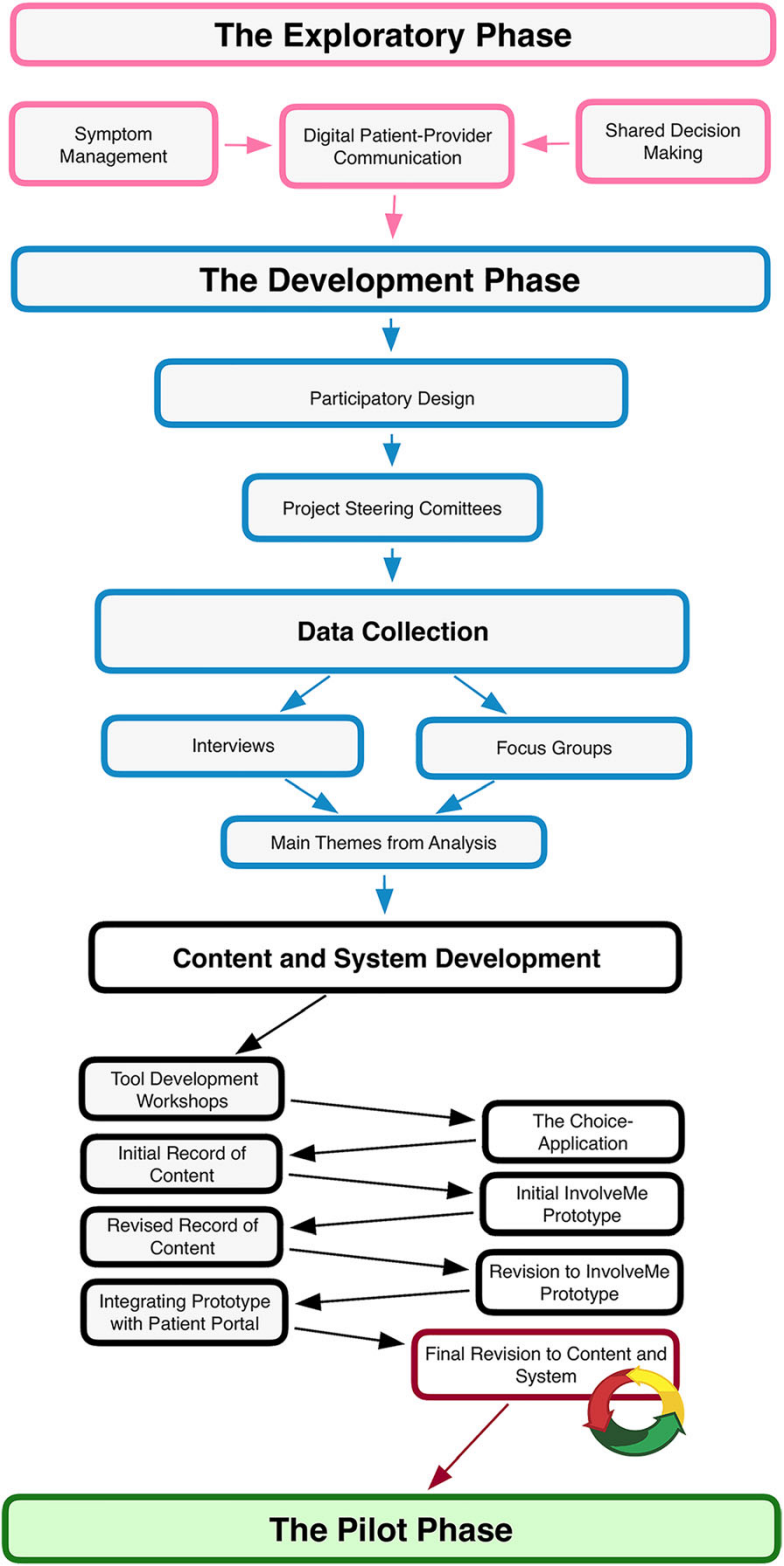
Overview of the development process

The current study is a qualitative study with a participatory design approach, where patients with chronic health conditions, HCPs, researchers and system developers collaborated closely [33]. Participatory design acknowledges the importance of including all stakeholders in the design process and promoting a common understanding of all perspectives during the process [34]. The data collection to map patients’ symptoms and needs was guided by existing reseach on symptom management in chronic conditions and advanced through patient interviews, focus groups with HCPs, and finally tool development workshops with patients and HCPs. The development process was informed by the data collection and guided by existing research on digital patient-provider communication and SDM. The final *InvolveMe* tool development was based on the *Choice-*application [24, 26, 29, 30] and integration with an existing patient portal, *MyRec* [35]. See Fig. 1 for an overview of the development process.

### Settings and participants

The participants in the current study were RTX or patients with NFPA, and the HCPs were recruited from either a nephrology- or an endocrine outpatient clinic at a large Tertiary Referral Center at a university hospital in Norway. Patients receiving follow-up at the clinics were invited to participate in the study. They had to be ≥18 years old and able to read and speak Norwegian. Both patient groups frequently experience a long period of slow deterioration of their health status before undergoing surgery, and both groups do experience a variety of symptoms in the aftermath [36–40], which negatively impact HRQoL [41, 42]. Renal replacement therapy through RTX is generally accepted as the preferred treatment for end-stage renal disease [36]. RTX symptoms include, but is not limited to, swelling, itching, thirst and changes in bowel and urinary habits [36]. Patients may also experience symptoms’ from medication side-effects and other co-morbid conditions [40]. NFPAs are benign pituitary tumors, and treatment is primarily surgery [37]. Patients face many challenges, included visual limitations, fear of recurrence, persisting distressing thoughts, loneliness and frustration [38, 39]. Both patient groups experience individuality and variability in symptoms, including pain, fatigue, sleeping problems, anxiety and depression [36, 38, 39]. Patients in both groups need and receive long-term follow up in outpatient care after surgery. HCPs in the current study were registered nurses, health support personnel, and physicians responsible for treatment and care for the respective patient groups.

### Stakeholder involvement

To strengthen the patient’s voice in the research process, the current study used the Guidance for Reporting of Patient and Public Involvement in Research (GRIPP2) prospectively to plan patient involvement [43]. Two patient representatives acted as advisors in the design and development process and contributed with refinement of written research material, interview guides and input for tool content development. The patient representatives were also members of the two Project Steering Committees established to promote involvement of all stakeholders. In addition, the head (physician) of each participating clinic, a registered nurse, a health support person and the first (BS) and senior (EB) authors participated in the Steering Committees. The Steering Committee’s role included decision-making regarding; planning and group facilitation approaches, recruitment and data collection strategies.

### Recruitment

Eligible patients were identified and informed about the study by registered nurses at the clinics. After consenting to be informed about the study, the potential participants were contacted by phone by a member of the research team who described the study and study participation in detail. Potential participants received oral as well as written information. HCPs at the participating clinics were also provided with information about the study. Those willing to participate were included in the study and informed consent was obtained from all participants.

### Data collection

Interviews with patients and focus groups with HCPs were conducted in order to map important symptoms and needs experienced by the patients groups. This informed the development of a digital patient-provider communication tool with opportunities for patients to self-report symptoms, needs and preferences for care prior to hospital visits, and secure messaging between hospital visits to support patients in the two separate patient groups (RTX and NFPA).

#### Interviews with patients (*n* = 14)

Participants completed a baseline demographic questionnaire related to age, sex, diagnosis, time since diagnosis and education. The goal was to collect information about the patients’ experience, understanding and perceptions of living with a chronic condition in order to map important symptoms and needs, as well as to gather information about patients’ expectations regarding digital communication. Interviews were directed by an interview guide (see Supplementary Material) focusing on these topics and conducted by the first author (BS), either face-to-face (*n* = 8) or by phone (*n* = 6). The interviews lasted between 20 and 75 min, were recorded with a digital voice recorder and transcribed verbatim. Participants received a gift card equivalent of USD 30 for their participation.

#### Focus groups with health care providers (*n* = 11)

Participants completed a baseline demographic questionnaire related to age, sex and years of work experience within the specialist health care setting. HCPs were invited to participate in focus groups to explore attitudes and experiences [44]. Two focus groups were conducted face-to-face by the first (BS) and senior (EB) authors. The HCPs from the nephrology- and the endocrine clinic participated in separate groups in order to develop patient group specific content. The interview guide consisted of open-ended themes in order to encourage discussion (see Supplementary Material). The themes focused on HCPs’ experiences with the types of questions patients often ask and appear to seek counseling for, as well as issues related to health technology. The focus group sessions lasted approximately 50 min, were recorded with a digital voice recorder and transcribed verbatim.

### Analysis

A thematic analysis as described by Braun and Clarke [45, 46] was conducted in order to identify, analyze and report patterns and themes from interviews (i.e., patients) and focus groups (i.e., HCPs). Themes generated from interviews and focus groups were compared to identify concordant and discordant perspectives. The analysis process was led by the first author (BS) in collaboration with the second and last authors (CV and EB). *The first step* in the analysis entailed becoming familiar with the transcripts. Brief notes were made of early impressions. In *step two*, data were organized into initial coding using the analytic software NVivo 11. All data considered relevant were coded. The codes were developed and modified through the continuous coding process. In *step three and four,* respectively, the main themes were generated and refined, and subthemes were identified for all main themes. *In the fifth step,* the wording of each theme and the overall story of the analysis were refined. Finally, in *the sixth step,* compelling quotes were selected and a final analysis based on the research questions conducted. The actual analysis process was more iterative than described, moving back and forth more than proceeding successively.

### Content and software development

The process of development was informed by the preceding data collection. The content and software development processes occurred in parallel, mutually influencing each other.

### Content development

Content development for the self-reporting of symptoms, needs and preferences part of the *InvolveMe* tool was informed by input from the data collection process. The self-reporting of symptoms, needs and preferences is collectively referred to as assessments. The data collection informed the development of separate assessments to suit each patient group (RTX and NFPA). Following data collection and analysis, four separate tool development workshops were conducted with patients and HCPs.

#### Workshops with patients (*n* = 6) and health care providers(*n* = 6)

Preliminary findings from interviews and focus groups were presented by the research team in tool development workshops with patients and HCPs in order to involve stakeholders in data evaluation and guide tool content development. To avoid patient-provider relationships influencing the discussions and to fit the content to each diagnostic group, two workshops with three patients each and two workshops with three HCPs each, were conducted. Workshop participants were invited to share their reflections through a card sorting exercise, a method that provides insight into how participants may expect organization of content to be [47]. Each card provided insight from interviews, focus groups and/or existing literature/guidelines [36, 38–42]. Insights from interviews included for example “fatigue” or feeling “depressed”, and insights from existing literature included for example “sleeping disturbances” or “sexual dysfunctions”. Participants sorted the insights into different themes and were encouraged to remove and/or add items as the workshop progressed. Input was also solicited regarding language and organization of content.

#### Software development

The *Choice* application contained a predefined list of symptoms that could be marked and graded for degree of bother and priorities for help [24, 26, 29, 30]. Based on options from *Choice*, the *InvolveMe* tool was developed and built to be integrated into an existing hospital patient portal, *MyRec* [35]. *MyRec* already had a secure login and options for secure messaging (email) between patients and HCPs, and the *InvolveMe* tool development therefore evolved around integrating the assessment functionality into *MyRec*.

The *InvolveMe* content and software development underwent several iterations, ensuring easily understandable language, brief and to the point sentences, and options for fitting content to the appropriate devices (e.g., content for small screens). The final selection of tool content was refined to fit a digital format and to facilitate easy and intuitive use. Software iterations were based on feedback (e.g., software features) from stakeholders as well as the constraints of *MyRec*, but were also affected by aspects from the content development (e.g., informational needs).

## Results

### Participant demographics

In interviews, the participating patients (*n* = 14) were median 54.5 years old (range: 37–67), with six women and eight men. Five patients had NFPA and nine were RTX. See Table 1 for patient demografics and clinical characteristics. In focus groups, six HCPs from a nephrology clinic and five HCPs from an endocrinal clinic participated in diagnostically separate focus groups. The participating HCPs (*n* = 11) were registered nurses (*n* = 6), health support personnel (*n* = 1), and physicians (*n* = 4), median 49 years old (range: 31–61) and had from two to 30 years of clinical experience from specialist health care.

**Table 1:** Patient demographics and clinical characteristics (*n* = 14)

Characteristics	median	(range)	*n*	%
**Age** (years)	54.5	(37–67)		
**Sex**				
Female			6	(43)
Male			8	(57)
**Diagnosis**				
Non-functioning pituitary adenoma^**a**^			5	(36)
End stage renal failure (Renal transplant recipient)			9	(64)
**Years with diagnosis**	8	(0.5–40)		
**Years since last surgery**	2	(0.5–17)		
**Education**				
Elementary/high school			2	(14)
University/college ≤4 years			7	(50)
University/college > 4 years			5	(36)

^**a**^One participant had not received surgery

### Results from interviews and focus groups

The findings from interviews with patients and focus groups with HCPs generated three main themes: 1) *Making symptoms and challenges visible*, 2) *Mastering a new life*, and 3) *Digital opportunities in follow-up*. See Table 2 for a thematic overview of content, and Table 3 for an overview of software functionalities.

**Table 2:** Thematic overview of content; Symptom and needs assessment in the *InvolveMe* tool

Bodily symptoms		
***Common for both patient groups***	***NFPA***^***a***^	***RTX***^***b***^
Pain	Menstruation	Breathlessness
Less interested in sex	irregularities	Swelling
Weakness	Hot flushes/night sweats	Itching
Dizziness	Erection changes	Bowel habit changes
Weight changes	Visual changes	Urinary habit changes
Fatigue	Coordination changes	Thirst
Sleeping problems	Balance changes	Nausea
**Psychosocial challenges**		
***Common for both patient groups***	***NFPA***	***RTX***
Anxiety	Stress sensitivity increased	N/A^c^
Depression		
Mood disturbances/fluctuations	Irritability increased	
Memory problems		
Concentration problems Lack of understanding (from		
family/friends)		
Lack of support (from family/friends)		
Guilt towards children		
Uncomfortable in social situations		
Loneliness		
**The need for work related support**		
***Common for both patient groups***	***NFPA***	***RTX***
Work-related understanding	N/A	N/A
Work disability		
The job exacerbates health		
**The need for information**		
***Common for both patient groups***	***NFPA***	***RTX***
Healthy diet	N/A	Infection prevention
Physical shape improvement		Skin/mole changes
Alcohol		
Smoking cessation		
Medications side effects		
Treatment options		
Disease progression		
Economic and social rights		
Medication prescriptions and certificates		

^**a**^*NFPA* Non-functioning pituitary adenomas

^**b**^*RTX* Renal transplant recipients

^**c**^*N/A* Not Applicable

**Table 3:** Overview of software functionalities in *MyRec* and the *InvolveMe* tool

MyRec’s existing functionalities	The integrated InvolveMe functionalities
Secure login and transfer of information	Digital invitation by HCPs to complete assessment prior to consultation
Secure message functionality	A predefined symptom and needs assessment
Available for HCPs on hospital computer	Grading of symptom severity
Patients as well as HCPs can initiate contact	Prioritizing symptoms and needs
Mail and text message notifications (patients/HCPs)	A generated summary based on patient priorities
	Previous assessments available
	Available for patient on smartphone/tablet/computer
	Designed for patient use in home settings

### Making symptoms and challenges visible

The participating patients expressed a need for a variety of symptoms to be addressed in the digital tool. Two subthemes were identified under this main theme; *Bodily Symptoms* and *Psychosocial Challenges*.

#### Bodily symptoms

Patients in both diagnostic groups described their experience with the physical aspects of their conditions, and that the symptoms and their impact often were or felt invisible to their HCPs. One patient stated:

***... you have to help patients on their way, particularly related to symptoms ... if you become accustomed to something, you might think it is normal...***

Some symptoms were diagnostic specific, and some were generic. One patient stated:

***...I often feel tired. I also have trouble sleeping. I sleep well when I finally fall asleep, but I do not fall asleep when I go to bed. So some days, I sort of- opt out. Simply because I feel so exhausted and tired.***

In the focus groups, HCPs described a number of symptoms often raised by the patients. For example, HCPs described weight and sexual changes as issues that were often raised by NFPA patients (these issues were barely mentioned in the interviews with patients). Similarly, HCPs for RTX patients explained that in addition to symptoms from the kidney disease, patients also experienced symptoms from co-morbidities.

#### Psychosocial challenges

Patients from both patient groups described similar experiences related to change of roles and negative impact on quality of life. One patient stated:

### Very often I say no to social activities

Some of the patients described struggling with emotions related to a changed life, while others described emotional concerns related to a failing health status or illness recurrence. One patient said:

***...if the blood samples are non-significant, it’s easy to forget how the patient actually feels...***

A few patients described experiencing difficulties accepting the physical changes caused by their illness, and described feeling alone and unsupported by their surroundings. Some patients described living alone as a source of vulnerability and anxiousness. NFPA patients also expressed vulnerability to stress and irritability. Those with responsibility for young children described worrying about how their illness was affecting their parenting. One patient said:

***... and then I'm a little scared for myself as I get really annoyed and angry, even with my own children, so I just have to leave the room, you just can’t really handle it there and then.***

In focus groups, HCPs for both patient groups described anxiety to be a common symptom for patients early in the disease trajectory, but that the degree of anxiety often decreased after surgery.

### Mastering a new life

This theme illustrates challenges that might occur after being diagnosed with a chronic condition. Because of changes along the disease trajectory, patients described constantly having to learn to manage new aspects of their condition. Two subthemes were identified: *The Need for Work Related Support* and *The Need for Information.*

#### The need for work related support

The participating patients described wanting to live a “normal” life. Those who were able to work wanted to work, even though they described experiencing limitations due to their illness and life situation. One patient stated:

***...I only work 50% and that's all I can do.***

Several patients described being unable to remain in a physically demanding job. Others described being able to go to work because they had an office job that did not require “too much”. Not being able to work full time, or not at all, was described as difficult. The patients also described at times needing practical support from their HCPs, their manager or from colleagues. As one patient stated:

***...it is a bit exhausting not to meet any understanding anywhere... I didn't receive any follow-up as a consequence …***

The need for work-related support was briefly brought up in the focus groups with HCPs.

#### The need for information

Many patients mentioned wanting to make lifestyle changes and described needing information about how to do so. Several gave examples about how they had experimented with diet, physical activity and alternative lifestyles. One patient stated:

***I have tried everything possible, I'm not on any medication you know...***

Patients with RTX described experiencing a new life with many medications which sometimes caused troublesome side-effects. Some stated that information from HCPs was not always useful because of “poor timing” or information not being tailored to their personal needs. Some patients described having experienced that HCPs sometimes hesitated to give them prognostic information. One patient said:

***I don't think one should always avoid things that are difficult to bring up or address.***

In the focus groups, HCPs stated that most NFPA patients were told by someone, prior to their hospital visits, that they had a tumor in their brain, which increased the patients’ level of anxiety. The HCPs described they were uncertain as to whether patients could find information on their own.

### Digital opportunities in follow-up

This theme illustrates challenges experienced by the patients when navigating the health care system, as well as potential opportunities for digital support and interaction. The main theme contained two subthemes; *Navigating the Health Care System* and *Digital Possibilities for Interaction.*

#### Navigating the health care system

Some of the patients had broad experience with the health care system through years of managing various condition related challenges, and expressed not always receiving adequate support. One patient stated:

***Because I contacted her [HCP], and she promised me that they would call me back, that she would discuss it with a physician, but then I never heard from her again.***

Patients described HCPs to be busy and difficult to contact by phone. A few patients said that if they could not reach their specialist by phone they would go to the hospital and wait until they could have an emergency consultation with their specialist HCP. One patient said:

***If I cannot reach them, after all I live less than two miles from the hospital … when I need to, I just go there.***

Patients were uncertain about where to find relevant health information from existing health portals. They described that it was common to forget questions, but said that it was also challenging to know who to ask, or what questions to ask. One patient said:

***I think you [HCPs] have to help people to know, that is, it’s not always that you know what you are wondering about... and then you don’t know what it’s okay to ask, or is it appropriate, you know, there’s a lot of self-censorship.***

In focus groups, HCPs described their daily work at the clinics as very hectic and that they sometimes forgot to document phone calls because of this. One HCP stated:

***Well, there are quite a few phone calls where you think you should have written things down, and then you have like ten calls in a row, so there’s information that simply gets lost in a way, cause you somehow just can't catch up.***

A few HCPs described sometimes sharing their private phone number with patients.

#### Digital possibilities for interaction

Most patients stated that it would be reassuring to use digital communication to contact their HCP. As one patient stated:

***Just to have a little more dialogue between all these treatments and visits … for you [HCPs] this is probably just day-to-day business and very common, but for me, it’s been the biggest crisis of my life, right? I have after all thought that I was going to die, or never become myself again, or become a vegetable. I’ve been through all of those, because yes, I know nothing about this stuff.***

Some patients suggested that an option to ask digital questions could prevent medication errors. As one patient said:

***I think that it can prevent mistakes, that maybe, that you receive some medicine you don't tolerate, or that you receive the wrong treatment, or that you don't get the follow-up you should have had.***

Patients also stated that digital contact with their health care provider could allow them to share what was important to them. One patient stated:

***This week I have had some serious issues with something or other, and then I write it down, and she [HCP] knows it. And then she can sit and think about it in advance, before I come for my appointment, and that’s really smart.***

Several patients stated that having “a digital list with symptoms” would be helpful, including using secure messaging with their HCPs.

Most of the participating HCPs wanted to communicate digitally with their patients, stating that such communication could potentially reduce the patients’ anxiety. HCPs also expressed concern that it might be difficult for patients to recall verbal information.“A digital list” would be useful for giving insight into patients’ needs and expectations prior to hospital visits, but HCPs stated that such a list should not be too extensive. HCPs also expressed the need to use an existing clinical system for digital patient-provider communication, and not an additional system that they would have to log into. As one HCP stated:

***We need to have fewer platforms to deal with, more functionality in the platforms we already use.***

### Content and software development

In line with the participatory design approach, potential future users (i.e., patients and HCPs) provided input on needs and requirements for the digital communication tool. The content and software development of the *InvolveMe* tool are presented below. The first two main themes from the data collection provided input for the development of the symptom assessment part of the *InvolveMe* tool. The third main theme gave input for development of software features for the *InvolveMe* tool, and supported the use of secure messaging between hospital visits to support patients.

### The InvolveMe tool content development

The data collection identified patient symptoms, needs and challenges, which were used to make a preliminary content record that was presented in the tool development workshops. Given that living with a chronic condition for many implies challenges related to concentration and fatigue, the research team made a decision that the content language should be brief and easy to read. See Table 2 for a thematic overview of tool content.

#### Workshops with participants

All patients participating in the tool development workshops were previously interviewed as a part of the initial data collection process. Four of the HCPs participating in the workshops had also participated in the focus groups, while two of the HCPs were “naïve” participants in order to elicit additional input. The workshops provided insight into how participants expected the organization of tool content to be, which provided input into the preliminary content record. At the same time, the workshops ensured that important topics only briefly mentioned in interviews with patients, potentially not yet addressed, were not left out. For example, the workshops with patients verified and elaborated on how psychological challenges and mental health were important factors to address for both patient groups. Similarly, workshops with both patients groups verified sexual dysfunctions as common. In addition, workshops with NFPA patients verified sleeping disturbances as essential to address.

#### Software development

**Fig. 2:**
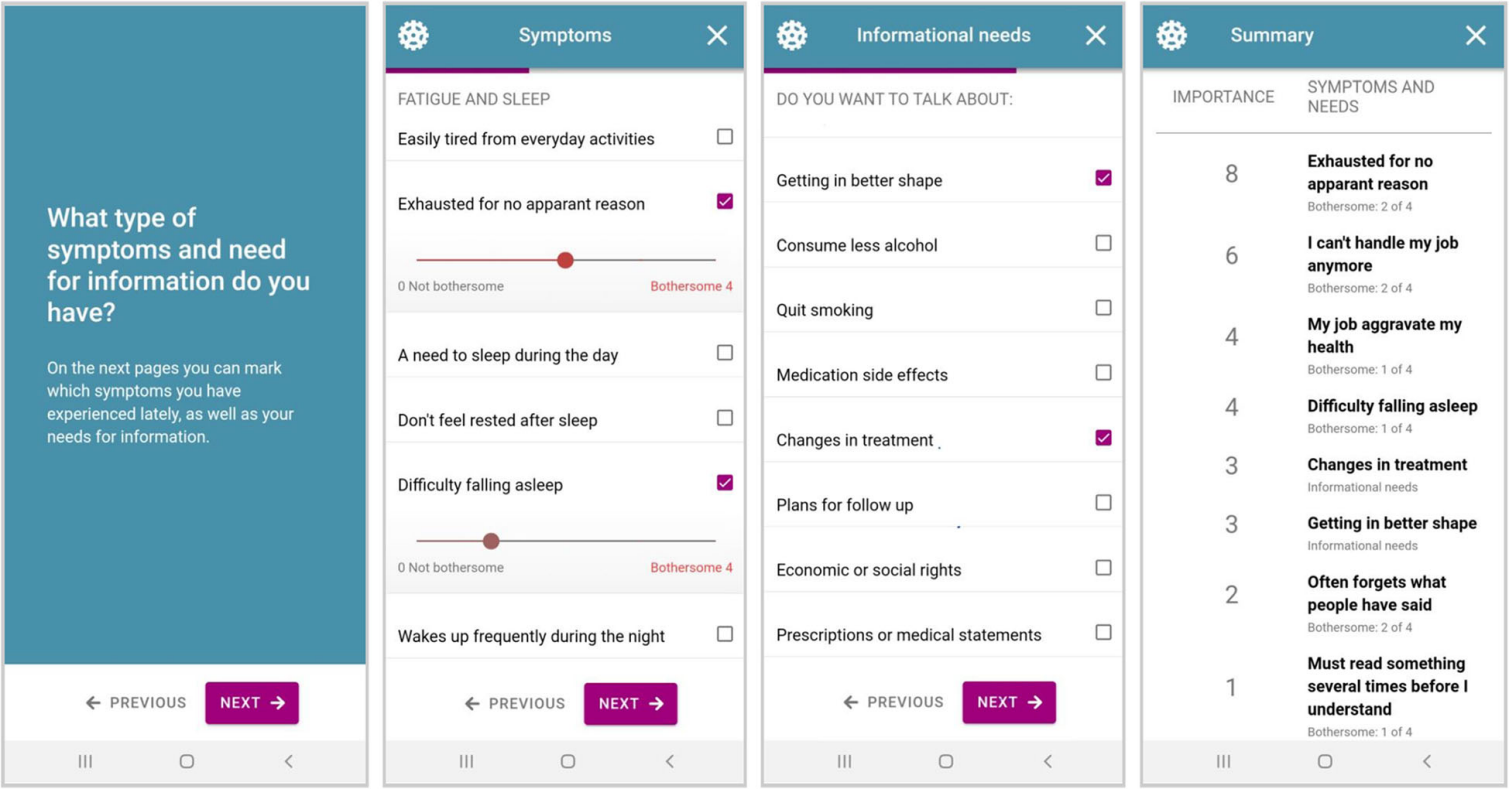
Screenshots from the patient interface of the *InvolveMe* tool

Based on stakeholder input, the research team (i.e., researchers and system developers) decided that the patient interface should be developed for smartphones or tablets, while the HCPs interface should be developed to fit hospital computers due to data protection legislation. The software development was also influenced by the content development process as the included content affected which features should be built. The assessment part of *InvolveMe* was built to be used by patients in their home settings, and for messages to be sent via the existing secure message functionality in *MyRec*. This would allow HCPs to invite patients to complete assessments prior to hospital visits, using system generated notifications by email or text message. The patients would then log into *MyRec* and open the email invitation to complete the assessment (i.e., a predefined list of symptoms and needs based on the thematic overview of content, see Table 2). The assessment included a functionality for rating how bothersome patients found current symptoms, and a function for prioritizing themes for discussion with their HCPs at the upcoming visit. By completing the assessment, the *InvolveMe* tool generates a summary. When completed and sent, the HCPs would receive a notification, the same notification would also apply for the use of secure messages between hospital visits. An overview of software functionalities is shown in Table 3. Screenshots from the patient interface of the *InvolveMe* tool are presented in Fig. 2.

## Discussion

### Principal findings

The current study describes the process involved in the exploration, data collection, content and software development of the *InvolveMe* tool; a patient-provider communication tool with two tailored symptoms and needs assessment versions suited to the symptoms and needs of patients with NFPA and RTX. Through the use of *InvolveMe*, patients will have the option to report their symptoms and needs, and rate how bothersome their symptoms are. *InvolveMe* will also provide patients with an opportunity to register their priority of themes to address in upcoming visit(s), and generate a summary intended for use in face-to face patient-provider communication during hospital visits. The integration of *InvolveMe* with *MyRec* provides options to complete the assessment at home prior to hospital visits on a variety of portable devices, as well as options to send secure messages between patient and HCPs.

### Describing patients’ current situation

Data collection from patients and HCPs revealed a wide range of symptoms and needs experienced by the participating patient groups. This is in line with previous research addressing the impact of living with chronic health conditions [1–4, 37–42]. Patients with chronic health conditions are characterized by large individual variations in symptoms, symptom frequency, symptom severity and the degree to which the patients are bothered by the symptoms, which makes it difficult to anticipate the type of care that may be in the patients’ best interest. Being able to communicate each patient’s current situation to HCPs can provide insight into patients’ clinical status and help identify important themes for patient-provider discussions [48], which is of essence for SDM [15]. Digitally reported measures can help patients describe the difficulties they experience [48], an issue described as challenging to convey by patients in the current study. The patients’ situation could also change from one hospital visit to another, making repeated assessments prior to each visit crucial for facilitating individually tailored care [13].

### Providing emotional support

The interviewed patients described facing many psychological challenges, a topic that was barely raised by the HCPs. Research has shown that if patients express emotional cues at all, these are often missed by the HCPs [49, 50]. Providing emotional support to patients when expressing negative emotions has been found to elicit clinically important information and foster patient-provider relationship [51]. Emotional support can also promote patient involvement, patient adherence to treatment plans, and other actions or behaviors that may lead to better patient outcomes [52]. The psychological symptoms described in the current study were included in the assessment part of the *InvolveMe* tool. Conducting an assessment prior to consultations has been seen to be an effective intervention in which patients can express cues and concerns, and also express such issues at an earlier point in consultations with their HCPs [53]. Facilitating a way for patients to communicate their symptoms to their HCPs has also been linked to reduced symptom distress [25, 26]. The fact that the *InvolveMe* tool includes assessment of psychological symptoms could therefore have the potential to facilitate patient-provider conversations where emotional symptoms and symptom distress are also included.

### Patient preference in symptom management

Including patient preferences can help set priorities for the hospital visit, and may facilitate patient-provider deliberation about symptom management, an essential part of chronic health care. Use of the original *Choice* application, compared with controls not using *Choice*, has been associated with significantly more symptoms being addressed in consultations [30]. Also, HCPs using *Choice* have reported that the tool served as a facilitator for mutual communication engagement between patients and HCPs, and also strengthened the patient voice and promoted shared care planning [27]. This is an important part of addressing the patient situation [17] and in line with SDM [15].

### Requesting information based on informational needs

Patients with chronic health conditions have been described as having difficulties interacting with their HCPs and understanding health information [5, 12]. Negative experiences may prevent necessary lifestyle adjustment or change, and may prevent patients from living well. The patient-provider relationship does in fact have a significant effect on healthcare outcomes [54], including influencing patients’ subjective symptom burden and HRQoL [55]. Assessing patients’ informational preferences can reduce patients’ informational needs and improve provision of information [55], which again indicates that information should be provided to patients at a point when they request such information. This mindset was incorporated into the *InvolveMe* tool by providing an opportunity for patients to request information based on present needs. Such features can potentially support patients in making choices about their lifestyle, on their own terms, when they are ready to do so. For example, patients using the *Choice* application reported feeling encouraged to ask more questions, and HCPs provided more information to their patients [30].

### Providing digital opportunities for patient-provider interaction

Patients**’** and HCPs**’** input on digital opportunities is in line with previous research showing that recall bias and inaccurate information can be reduced by using digital communication [48]. In the chronic health care setting, patient-provider communication through secure messaging can act as a valuable supplement to standard care [23, 28, 32], supporting patient-provider relationships through enabling reassurance and commitment for the patient as well as the HCP. Research also suggests that patient access to use secure messaging with HCP may be linked to reduced depression among patients with serious illness [27]. Access to digital patient-provider communication have been seen to provide patients with a sense that the HCPs are interested in them and more present, an increased sense of connection between patients and HCPs, and also a reduced sense of being alone in dealing with their condition for the patients [48]. Through increasing two-way communication and thereby strengthening the patient-provider relationships, an essential part of SDM [15], secure messaging clearly has the potential to be a powerful tool in the management of chronic health conditions.

### Study limitations, strengths and future directions

This study has several limitations. First, two separate patient groups were included in the development process of the *InvolveMe* tool. Differences between the two groups could potentially complicate the development process. However, as shown in Table 2, the two patient groups shared most symptoms and expressed needs. Second, the use of an existing application (i.e., *Choice*) and integration with an existing patient portal (i.e., *MyRec*) may have limited the design and development process, including potentially reducing creativity. However, the development process of *InvolveMe* was based on a digital intervention already proven to have effect [24, 26, 29, 30, 53, 56], and integrated with an existing patient portal already used by HCPs, a combination that likely will support implementation. Finally, the *InvolveMe* tool does not use validated outcome measures to obtain symptoms and needs data, which may raise questions as to whether *InvolveMe* measures as intended. However, the *InvolveMe* tool was not designed to be a validated measure of symptoms, but rather to provide options for patient-provider interaction, and to improve and individualize patient-provider communication in long term follow up of patients with chronic health conditions.

This study also has several strengths. The development process took place in close collaboration with stakeholders, including patients, HCPs, researchers, system developers and patient representatives. Through stakeholder involvement of potential future users, from study initiation through exploration, design and development processes, the study ensured inclusion of content and software functionalities relevant and meaningful for patients as well as HCPs. Such a process has the potential to contribute to increasing the potential effectiveness of the developed intervention. Another strength of the current study is that multiple data collection methods with stakeholders directly informed the selection and refinement of the symptom and needs assessment part of the *InvolveMe* tool.

Future studies should investigate the perspectives of patients as well as HCPs regarding the usefulness and feasibility of the *InvolveMe* tool in clinical practice. To explore usability and potential effects of the *InvolveMe* tool, a feasibility pilot study is in progress where patients as well as HCPs will test content and software functionalities in a clinical context. In the upcoming pilot study, exploring how use of the *InvolveMe* tool affects the patient-provider interaction and communication will be of essence.

## Conclusion

The current study described the participatory design approach, incorporating stakeholder input, evidence and theory, when developing the content and software of *InvolveMe*, a patient-provider communication tool for chronic health care settings. The *InvolveMe* tool has the potential to strengthen patient-provider partnership and improve communication by drawing out what matters to each individual patient. The use of the tool should be regarded as a supplement to standard care. The described design and development approach, aiming to improve and individualize patient-provider communication in long term follow up may provide input for other researchers aiming to develop digital interventions in chronic health care settings. While the usability, feasibility and hypothetical efficacy remains to be tested, a tool such as *InvolveMe* may have the potential to be useful for a variety of patient groups with chronic health conditions.

## Supplementary information

**Additional file 1.** InvolveMe Interview guide

## Data Availability

The datasets used and/or analyzed during the current study are available from the corresponding author on reasonable request. Access to the patient portal *MyRec* is available at; *https://L2W.no/ytbe*

## Abbreviations

GP: General physician
HCP: Healthcare provider
HRQoL: Health Related Quality of Life
NFPA: Non-functioning pituitary adenoma
RTX: Renal transplant recipient
SDM: Shared decision making
