# Supplementary material for: Content and system development of a digital patient-provider communication tool to support shared decision making in chronic health care: InvolveMe
Source: BMC Med Inform Decis Mak. 2020 Mar 4;20:46. doi: 10.1186/s12911-020-1065-8 (PMC7057594; doi:10.1186/s12911-020-1065-8)
Supplement: Supplementary file 1 — Additional file 1. InvolveMe Interview guide [file 12911_2020_1065_MOESM1_ESM.pdf]

InvolveMe
Interview guide
Patients

Theme: Development of a digital communication tool
We aim to develop a digital patient- provider communication tool. The tool should be used prior to hospital visits as a preparation for consultations for both patient and health care providers. Also, it should provide the opportunity for follow-up between consultations.

1. Can you talk about the course of the disease and your current health situation?

2. Can you talk about how consultations with health care providers usually take place today?

3. Can you talk about what you wish was different in the follow-up from health care providers?

4. Can you talk about how you keep track of symptoms and illness today?

5. How do you use technology in everyday life, and do you use it to monitor your own illness/symptoms ?

6. Can you tell us what requirements and expectations you have for the content and use of a digital communication tool?

In conclusion:

7. Is there anything that is particularly important that we should keep in mind when we start tool development?

InvolveMe
Focus group Interview guide
Health care providers

Theme: Development of a digital communication tool
We aim to develop a digital patient- provider communication tool. The tool should be used prior to hospital visits as a preparation for consultations for both patient and health care providers. Also, it should provide the opportunity for follow-up between consultations.

1. Can you describe the patient group?

2. Can you talk about how consultations are carried out today?

3. Can you talk about what impression you have on how patients themselves monitor disease progression and symptoms today?

4. Is there anything that you wish could be different regarding the follow-up of the patient group?

5. Can talk about how technology is used in patient follow-up today?

6. Can you talk about what kind of requirements and expectations you have for content and usability of a digital communication tool?

In conclusion:

7. Is there anything that would be particularly important for us to keep in mind when starting tool development?
